# Alcohol Withdrawal and Brain Injuries: Beyond Classical Mechanisms 

**DOI:** 10.3390/molecules15074984

**Published:** 2010-07-20

**Authors:** Marianna E. Jung, Daniel B. Metzger

**Affiliations:** Department of Pharmacology and Neuroscience, University of North Texas Health Science Center at Fort Worth, 3500 Camp Bowie Blvd., Fort Worth, TX 76107-2699, USA; E-Mail: Daniel.Metzger@unthsc.edu (D.B.M.)

**Keywords:** antioxidant, brain aging, 17β-estradiol, ethanol withdrawal, mitochondria, oxidative stress, protein kinase, protein oxidation

## Abstract

Unmanaged sudden withdrawal from the excessive consumption of alcohol (ethanol) adversely alters neuronal integrity in vulnerable brain regions such as the cerebellum, hippocampus, or cortex. In addition to well known hyperexcitatory neurotransmissions, ethanol withdrawal (EW) provokes the intense generation of reactive oxygen species (ROS) and the activation of stress-responding protein kinases, which are the focus of this review article. EW also inflicts mitochondrial membranes/membrane potential, perturbs redox balance, and suppresses mitochondrial enzymes, all of which impair a fundamental function of mitochondria. Moreover, EW acts as an age-provoking stressor. The vulnerable age to EW stress is not necessarily the oldest age and varies depending upon the target molecule of EW. A major female sex steroid, 17β-estradiol (E2), interferes with the EW-induced alteration of oxidative signaling pathways and thereby protects neurons, mitochondria, and behaviors. The current review attempts to provide integrated information at the levels of oxidative signaling mechanisms by which EW provokes brain injuries and E2 protects against it.

## 1. Ethanol Withdrawal (EW)

EW refers an abrupt termination of long-term heavy ethanol consumption, the acute syndromes of which range from psychological symptoms to physical signs. Those are anxiety, depression, tremors, rigidity, hyperactivity, convulsion, coma, and even death. These signs often determine whether or not an individual is alcohol dependent. Many alcohol dependent individuals attempt to quit ethanol abuse but distress associated with these syndromes provides motivation to relapse into ethanol abuse [1]. As they repeat the vicious cycle of hazardous drinking and unsuccessful detoxification, the intensity of EW syndromes increases [2]. Clinically, benzodiazepines are the primary available treatments but their side effects such as respiratory inhibition or dependence liability limit their therapeutic usage. As a step towards the development of a better research and therapeutic strategy, we and others have strived to identify the mechanisms by which EW damages the brain. 

Numerous studies have documented deleterious effects of ethanol abuse such as liver damage, increased inflammation, cardiac dysfunction and cognitive deficit [3,4]. However, compared to ethanol *per se*, relatively few studies have reported the effects of EW on the brain. EW stress is initiated by a sudden removal of a neurosuppressant ethanol. Therefore, classical mechanisms underlying EW stimulus involve enhanced excitatory (e.g. glutamate) and suppressed inhibitory (e.g. GABA) neurotransmittal systems [5]. These mechanisms, as well as other neurotransmittal systems such as dopamine and serotonin involved in ethanol/EW effects, were thoroughly reviewed by Fadda and Rossetti [5] and by Jaatinen and Rintala [6]. However, beyond the neurotransmittal mechanisms, a body of evidence revealed that EW stimulates the pathways of unstable oxidative molecules and stress-responding signaling proteins (e.g. PKC and P38). Unfortunately, many alcohol studies did not differentiate between ethanol exposure and EW and thus it is not clear whether the reported injuries/mechanisms are associated with ethanol toxicity, EW, or both. In order to differentiate the effects of EW from those of ethanol exposure, tissues or cells should be collected in the optimum hours after ethanol is completely eliminated from blood or cell cultures. The optimum hours vary depending upon an ethanol regimen. In our model of EW, severe neurochemical abnormalities were observed in the brains harvested 24 hours after the termination of chronic ethanol consumption (35 or 90 days) or in cells withdrawn for 4 hours from 24 hours-ethanol exposure. EW-induced seizure was observed 1 or 3 days after the cessation of an ethanol diet for 14 days [7]. The differentiation between ethanol and EW stress is important because the deleterious effects of EW are not necessarily identical to those of ethanol *per se* and can cause more brain damage. In support of this idea, ethanol treatment followed by withdrawal resulted in a neuronal loss in the mouse hippocampus [8] and rat cerebellum [9], whereas ethanol *per se* did not cause any neuronal loss. EW but not ethanol exposure resulted in shrinkage and disintegration of neuritic processes and cell bodies in primary cortical neurons [10]. Similarly, EW but not ethanol exposure decreased the cyclic AMP-response element-binding proteins, a Ca^2+^-dependent kinase in the cortex [11], and a Ca^2+ ^buffering protein parvalbumin [12]. Repeated EW, but not an equivalent amount of continuous ethanol exposure, impaired the learning ability associated with amygdale [13,14]. In line with these greater disturbing effects of EW than ethanol, De Witte et al. [2] stated that neuroadaptations occur within the CNS during alcohol dependence, allowing the brain to function regularly while being disturbed by alcohol. We did observe that although both ethanol and EW produced oxidative damages to lipids and proteins in rats and cells [15,16], the degree was more severe during EW, suggesting that EW is distinct from ethanol exposure *per se*.

## 2. EW & Oxidative Stress

### 2.1. Oxidative stress

Reactive oxygen species (ROS) are produced under physiological conditions as byproducts of redox reactions but the endogenous antioxidant defense system usually scavenges them. However, under pathological conditions, the excessive generation of ROS overcomes the defense system and triggers the cascade of deleterious molecular events called oxidative stress. A moderate generation of ROS can be beneficial for cell survival by enhancing adaptive mechanisms [17,18]. The current review article discusses mainly a deleterious degree of oxidative stress. Oxidative stress-induced neuronal damage has been implicated in neurodegenerative disorders including those associated with ethanol consumption and EW. While ethanol itself directly generates ROS during its metabolism, EW produces oxidative stress indirectly through the excitatory neurotransmitter system or excitatory molecules such as Ca^2+^ [19]. EW-induced ROS subsequently promote lipid peroxidation which reflects the interaction between oxygen and the polyunsaturated fatty acids of membrane lipids (e.g. malondialdehyde), further generating deteriorating breakdown products. 

A functional impairment attributed by pro-oxidant EW was seen in a study in which enhanced malondialdehyde concurred with the enhanced severity of EW syndromes [20] such as seizure activity [21,22,23]. The pro-oxidant nature of ethanol or EW is particularly important in the CNS because CNS consists of a high content of unsaturated membrane lipids, which are a preferred target of both ROS and lipid peroxidation [24,25]. In addition, despite that the brain has a high oxidative metabolic rate, it has low levels of antioxidant enzymes compared to other organs [26]. We did observed that excessive lipid peroxidation occurred in the ethanol withdrawn rat cerebellum and this phenomenon was more severe in the membrane fraction than the cytosol fraction [27]. These studies suggest that the membranous structure of brain provides a favorable condition to EW insults. The membrane targeting effect of EW is also seen in a clinical situation in which the membrane fluidity of erythrocytes was altered in ethanol withdrawn alcoholic patients [28]. EW-induced oxidative stress appears to be transient under a certain condition. Marotta *et al*. [29] stated that the oxidative phenomena occur in the early phase of EW rather than the late phase. In agreement, an increased level of malondialdehyde during early detoxification in alcoholic patients decreased within a few days [20,30]. In our animal study, lipid peroxidation peaked at 24 hours of EW and diminished toward the control level after 2-4 days of withdrawal [27]. Such transient oxidative insults may not immediately result in neuronal death because no TUNEL (marker of DNA fragmentation) positive cells were detected at 48 hours of EW (data not shown). Ulrichsen *et al*. also showed no hippocampal neuronal loss during the early EW phase after multiple EW events in rats [31]. Although the levels of these oxidative markers are decreased as the day of EW elapses, the significantly elevated levels of oxidative molecules during the acute phase of EW may trigger downstream adverse effectors, ultimately contributing to neuronal damages. In fact, rat cerebellum obtained two weeks after the termination of an ethanol diet (five weeks, 6.5% v/v/) showed a loss of Purkinje neurons [9]. The EW-induced neuronal damage may be promoted by an excitatory neurotransmission such as the glutamatergic system based on a report that the inhibition of glutamate transporters (terminator of glutamatergic neurotransmission) generated ROS in the rat hippocampus [32]. Therefore, it is possible that an additive or a synergistic effect of ROS with glutamate may exacerbate the neuronal loss. Indeed, Coyle and Puttfarcken [33] stated that the persistent activation of glutamate-gated ion channels and oxidative stress are interacting processes which provide a final common pathway for cell vulnerability in the brain. On the other hand, one cannot exclude a possibility that the levels of oxidative markers may remain elevated for longer than a few days depending upon the severity of EW or the type of brain injuries. 

### 2.2. Protein oxidation

The pro-oxidant effects of EW are also seen at the protein level. Protein oxidation is particularly important because neurodegenerative diseases and aging are associated with the oxidative modification of proteins [34,35]. ROS attack proteins, resulting in the formation of protein carbonyls and subsequently inactive proteins. Therefore, augmented carbonyl contents have been measured as a cellular marker of oxidative damage to proteins [36,37]. The protein carbonyl content was increased in the blood of ethanol-dependent patients [38] and in the liver or pancreas of ethanol exposed rats and mice [39,40,41]. Similarly, an increased content of protein carbonyls was observed in the cerebellum, cortex, and hippocampus of ethanol consuming rats [16]. Importantly, the level of protein carbonyls was further increased during EW [16], suggesting that abrupt EW produces more severe protein oxidation than ethanol *per se*. 

The mechanisms by which EW provokes oxidative stress/damage have been characterized in our *in vitro* immortalized hippocampal cell line (HT22 cell). This cell line is an effective model of oxidative stress. HT22 cells contain the glutamate/cystine antiporter for the delivery of cystine into neuronal cells that mediates the synthesis of an antioxidant glutathione. Therefore, HT22 cellular injury is often associated with oxidative insults [42,43] including EW-induced cellular oxidation [44]. As was the case for *in vivo* studies, HT22 cells withdrawn for four hours after ethanol exposure for 24 hours encountered an excess generation of ROS and protein carbonylation. The oxidative stress was more severe during EW than ethanol exposure *per se* and was accompanied with cell death. Taken together, these findings suggest that EW induces destructive oxidative stress both in *in vivo* and *in vitro* conditions. 

### 2.3. Antioxidant mechanisms of E2

**Figure 1:**
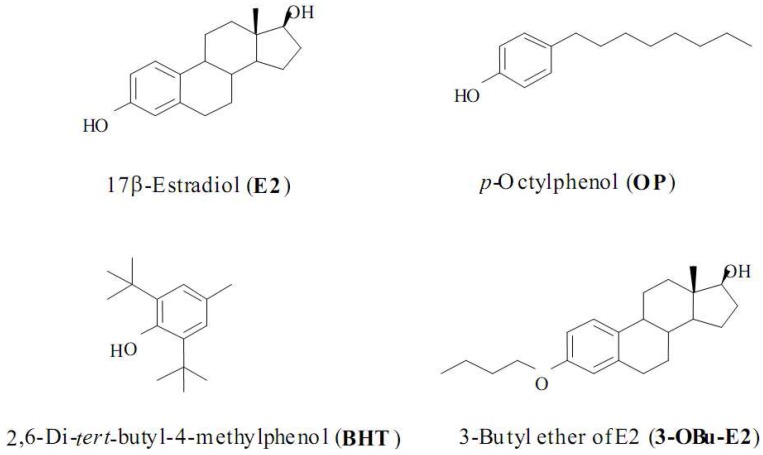
Chemical structures of E2, OP, BHT and 3-OBu-E2.

**Figure 2:**
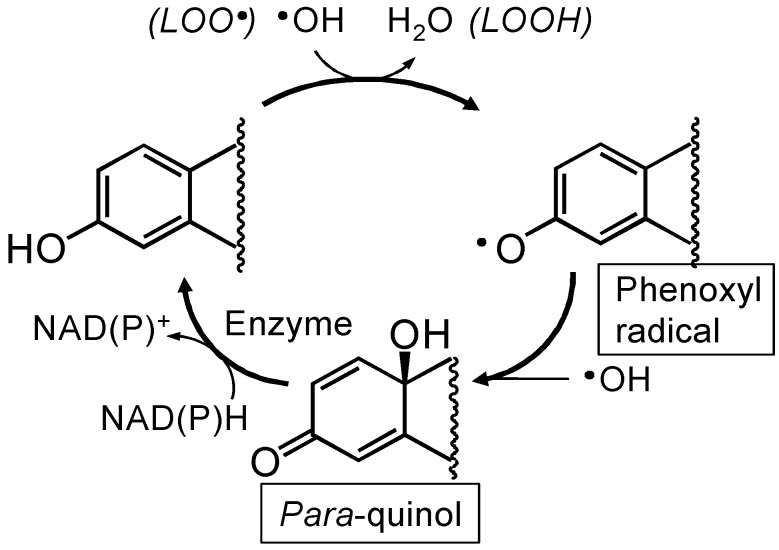
Schematic illustration of how estrogens provide a “chemical shield” from •OH exposure by an antioxidant cycle discovered by Prokai [54,55]. E2 captures •OH and produces the phenoxyl radical and subsequently a bioreversible quinol. The quinol is rapidly converted to the parent estrogen via a NAD(P)H-dependent enzyme-catalyzed reductive aromatization to perpetuate the antioxidant action. During this process, •OH is detoxified to H_2_O via a radical exchange reaction in the chain-breaking lipid peroxidation (LOO· → LOOH).

17β-Estradiol (E2, Figure 1) is the most potent naturally occurring estrogen. Beyond its effect on reproductive organs, the neuroprotective activities of E2 have been extensively studied and proved in a variety of *in vitro* and *in vivo* neuroprotection models [45,46,47,48,49,50]. The E2’s neuroprotection is the end results of well-orchestrated genomic and non-genomic processes. As a non-genomic protection, the estrogen receptor (ER)-independent antioxidant effect of E2 is an important contributor to cell survival against oxidative stress [51,52,53]. The antioxidant action occurs mainly via direct free-radical scavenging, although indirect mechanisms, such as the upregulation of antioxidant enzymes and the chelation of redox-active metal ions, may also be involved [54,55]. E2 may directly scavenge free radicals through a mechanism involving a non-phenolic para-quinol from which E2 can be efficiently regenerated via a NADPH-dependent reductive aromatization (Figure 2) [53,54]. This cyclic antioxidant mechanism discovered by Prokai implies that E2 doesn’t have to be used up when it provides a “chemical shield” against harmful free radicals but it can be rejuvenated. Indeed, E2 protected against the excessive generation of ROS in HT22 cells which were inflicted by a side effect of a psychiatric treatment [56].

E2 also mitigated EW-induced lipid peroxidation and protein oxidation in rats and cells [16,57]. Estrogen protection against EW-induced oxidation may also involve the regulation of glutamatergic neurotransmission because glutamate-induced oxidative stress was attenuated by E2 [58,59]. Additionally, E2 may counteract pro-oxidant ethanol/EW through enhancing the activity of antioxidant enzymes based on a report that estrogen deficiency downregulated the activity of superoxide dismutase [60]. The downregulation of antioxidant enzymes is associated with increased production of free radicals and is prevented by estrogen replacement [60]. Furthermore, estrogens at a physiological concentration interact synergistically with the antioxidant glutathione, providing enhanced neuroprotective potency [61,62]. This synergy with glutathione may result from an enhancing effect of estrogen on the reducing potential of glutathione [59,63]. 

We recently assessed whether a free radical scavenging effect of estrogen contributes to its protection against pro-oxidant EW in HT22 cells [64]. An *in vitro* efficacy of E2 was compared with other phenolic compounds such as *p*-octylphenol (OP) and 2,6-di-*tert*-butyl-4-methylphenol (BHT) and a non-phenolic compound, 3-butyl ether-E2 (3-OBu-E2) in HT22 cells. 3-OBu-E2 is an E2 derivative (Figure 1) in which a phenolic OH is blocked by butyl ether formation [65]. If the antioxidant property of these compounds is attributed to the presence of the free phenolic OH, 3-OBu-E2 should have no protection against EW-induced oxidation. As expected, E2, *p*-octylphenol, and BHT protected against EW-induced cell death and cellular oxidation whereas 3-OBu-E2 without the phenolic OH failed to exert the protection (Table 1). These findings suggest that the phenolic moiety of E2 is, indeed, an essential feature for the free radical scavenging property. 

**Table 1:** The comparison between protective effects of phenolic and non-phenolic test compounds. HT-22 cells were exposed to 24- hour-ethanol (200 mM) followed by 4-hours-ethanol withdrawal (EW) with or without (EW only) concomitant treatment with phenolic test compounds (1µM, Figure 1) [15,64]**. **In control experiments (vehicle only, no ethanol), cell viability was counted as 100%, MDA level was 173 ± 6.5 nmol/mg℘protein and protein carbonyl content was 1.28 ± 0.01 nmol/mg℘protein.* p < 0.01 *vs.* corresponding EW only (no test compound).

% of control	EW only	EW + Test Compound (1 µM)			
		E2	BHT	OP	3-OBu-E2
Cell survival	30 ± 1	49 ± 2*	50 ± 1*	48 ± 3*	28 ± 3
MDA content	219 ± 2	157 ± 3*	152 ± 1*	161 ± 3*	220 ± 2
Protein carbonyl	229 ± 3	171 ± 3*	174 ± 2*	175 ± 2*	230 ± 3

## 3. Protein Kinase

### 3.1. PKC

A body of evidence indicates that oxidative stimuli are closely linked to signaling protein kinases such as protein kinase C (PKC) [65,66]. PKC is an important regulatory enzyme in cell fate and phosphorylates a variety of substrates such as transcription factors, membrane receptors, ion channels, and nuclear proteins. Although many functions of PKC are beneficial for cell survival [67,68,69], prolonged or excessive PKC activity can promote adverse downstream events [70].

The PKC family is comprised of approximately 10 different isozymes (α, βI, βII, γ, δ, ε, θ, η, ζ, and ι/λ) depending upon structural features and requirements for PKC activation [70]. Among these isozymes, PKC epsilon (PKCε) has been particularly implicated in the effects of ethanol/EW effects. A recent *in vitro* study identified an ethanol-binding site in PKCε [71]. Mutant mice lacking PKCε consumed less ethanol and displayed less severe EW-induced seizure than wild-type mice [72]. Ethanol exposed cultured neurons showed an increase in the level of PKCε [73]. Acute ethanol treatment enhanced the activity of PKCε in the cardiac muscle [74], whereas chronic ethanol treatment decreased the activities in the cortex and hippocampus [75]. Withdrawal from forced intermittent ethanol administration for seven days enhanced the activity and protein levels of PKCε, whereas withdrawal from voluntary ethanol consumption for five weeks suppressed PKCε activity [76]. As such, the effect of ethanol or EW on PKCε may vary depending upon the regimen of ethanol treatment. Nevertheless, these studies suggest that PKCε is involved in the stimulus effects of ethanol dependence and EW. PKC activation may require the stimulation of resting/inactive PKC in the cytosolic compartment and its relocation to the membrane component of cells [77,78]. It has been demonstrated that ethanol exposure and EW increase the membrane translocation of PKCε [73,79]. In our previous observations, both ethanol exposure and EW increased membrane translocation and membrane protein levels of PKCε but only EW enhanced the activity of PKCε. This suggests that membrane translocation is pre-requisite but not sufficient for PKC activation. Presumably, PKC, after translocation to membrane, may have to bind to specific anchoring proteins for functional activation [80]. This process may be completed during EW but not during ethanol exposure *per se*. 

The protective effects of estrogen on PKCε are controversial. PKCε mediates hyperalgesia caused by inflammation [81] and by chronic alcoholism [82] in a manner that was attenuated by E2 in female rats [83]. By comparison, estrogen alone without any stressful stimuli activated and induced the translocation of PKCε to the plasma membrane of cultured dorsal root ganglia neurons [84]. In our EW model, estrogen protected the homeostatic status of PKCε upon EW stress [76] and glutamate-induced cytotoxicity [85]. The mechanism(s) through which estrogen inhibits or activates PKC activity are unclear. One can speculate that an antioxidant effect of E2 may interfere with a pro-oxidant pathway associated with PKC. At concentrations that alter the intracellular distribution and activity of PKC, E2 demonstrates potent antioxidant properties in HT-22 cells [86]. Oxidants selectively react with the regulatory domain to activate PKC whereas antioxidants appear to interact with the catalytic domain to inhibit cellular PKC activity [87]. Presumably, while pro-oxidant EW activates PKC and induces the membrane translocation, the antioxidant activity of E2 stabilizes neuronal membranes, inhibits the binding of PKC to anchoring proteins, and thus prevents the functional activation of PKC [80]. Further studies need to elucidate precise mechanisms underlying the dual effects of E2 on PKC. 

### 3.2. P38

In addition to PKC, P38 is intimately involved in mediating EW insults. P38 belongs to the family of mitogen-activated protein kinases that mediate signaling cascades and regulate cell fate in response to cellular stress [88]. As is the case for PKC, a transient, moderate activation of P38 is associated with cell survival or differentiation, whereas a sustained or excess activation generally correlates with pathological conditions [89,90,91]. P38 is activated upon phosphorylation [92] and thus, pP38 is often measured as an indicator of P38 activation. Whether or not PKC (PKCε) is linked to a P38 pathway is controversial. While the overexpression of PKCε did not alter P38 activation in one study [93], a downregulation of PKCε blocked P38 activation in another study [94]. In either case, P38 is phosphorylated in response to stress signals such as inflammatory cytokines, heat shock or ischemia [95]. Accordingly, P38 is known as a stress-activated protein kinase. The P38 family minimally includes P38α [96], P38β [97,98], P38γ [99,100] and P38δ [101]. Among these isozymes, P38α and P38β are highly expressed in brain areas that are vulnerable to ethanol/EW, such as cerebellum and cortex [102,103,104]. 

The signaling role of P38 in the effects of ethanol has been demonstrated such that P38 inhibitor SB203580 attenuated ethanol-induced HT22 cell death [105]. Acute ethanol treatment provoked P38 activation [106] and augmented endotoxin-induced pP38 levels in human monocytes [107]. Our recent study identified that P38 is activated more severely during EW than during ethanol exposure in male and female rats [108]. Because P38 activation has both beneficial [109] and deleterious [110] effects, we tested the effects of EW-induced P38 activation on cell survival. When P38 inhibitor (SB208035) was administered during EW, HT22 cells survived from EW-induced cytotoxicity. By comparison, the protection was lost when SB208035 was administered during ethanol exposure *per se*. Given this, it appears that an excessive activation of this kinase is toxic to cells and EW-induced rather than ethanol-induced activation of P38 contributes to the EW cytotoxicity. 

It is not well understood how EW provokes P38 activation. As one possible mechanism, EW-induced glutamate neurotransmission may be involved in this process based on a study in which glutamate increased P38 phosphorylation in cultured chick cerebellar glial cells [111]. Another potential mechanism is inferred from studies in which oxidative stress was attributed to P38 activation [112,113]. When SB203580 (P38 inhibitor) treatment was restricted to the EW phase, it attenuated ROS generation in HT22 cells. These studies suggest that EW induces a deleterious interaction between oxidative pathways and P38 activation, contributing to cellular and neuronal damages. 

**Figure 3:**
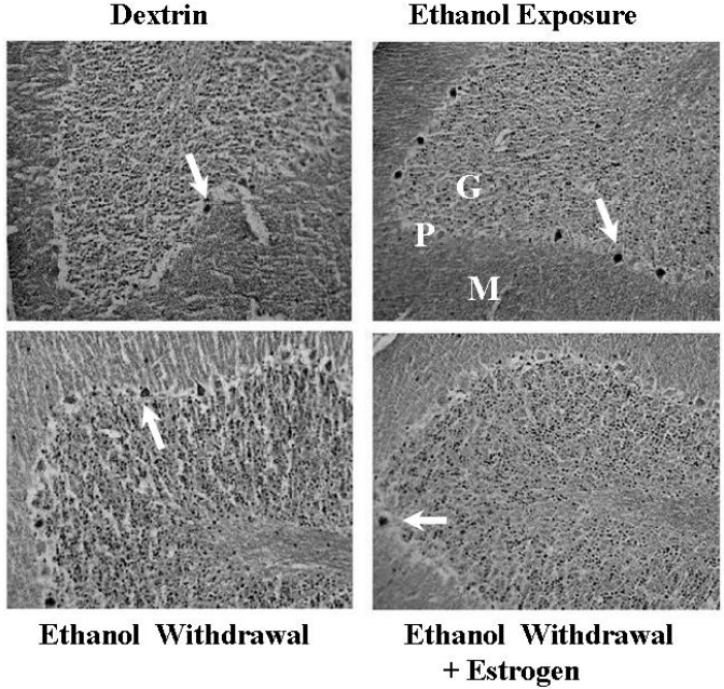
Immunohistochemical detection of pP38 expression in the cerebellum. Ovariectomized rats implanted with oil pellets or E2 pellets began a 25-day-ethanol (6.5%) and 5-day-withdrawal, repeating 3 times. The ethanol-exposure group continuously received an ethanol-diet until the very end of a diet program and was gradually withdrawn. At the 14th day of the last EW, the left hemisphere containing the cerebellar vermis was processed for immunohistochemical analysis to locate pP38 protein. Dark deposits marked with arrows indicate pP38 positive neurons or cells in the Purkinje (P) and granular (G) layers. A magnification of a 20-fold was used to take pictures. A scale bar indicates an actual length of 200 μm.

Protective effects of E2 are also shown at the level of P38. E2 attenuated the phosphorylation of P38 induced by angiotensin II in cells [114], cardiac hypertrophy in ovariectomized mice [115], and myocardial inflammation in ovariectomized rats [116]. Valles *et al*. [113] suggested that E2 prevented oxidative stress, which in turn inhibited P38 activation and protected neurons from Amyloid β-toxicity. In our recent study, EW increased the number of cerebellar Purkinje neurons containing pP38 and E2 treatment decreased it toward a control level (Figure 3). In contrast, trauma-hemorrhage decreased the protein level of pP38, and E2 treatment increased it toward a control level [117]. It is not clear what mediates the inhibiting and the activating effects of E2 on P38. At the very least, E2 may modulate the pathological conditions of P38 in a direction toward the homeostatic status of P38. 

## 4. EW & Mitochondria

The primary function of mitochondria is to produce cellular energy (ATP) through a series of mitochondrial enzyme complexes located in the inner mitochondrial membranes. Electrons are transferred across the enzyme complexes and create the electrochemical gradient between the mitochondrial membranes. Subsequently, the electrochemical gradient provides a force to generate ATP. In the process of the electron transfer, ROS such as superoxide are typically generated as byproducts. However, the levels of ROS increase dramatically under certain pathological conditions such as EW, resulting in significant cellular and neuronal damages. 

Exogenously applied H_2_O_2_ increased mitochondrial protein oxidation in conjunction with ATP depletion in cultured human fibroblasts [118] and human lens epithelial cells [119]. Acute administration of ethanol (5 g/kg, IP) to mice depleted mitochondrial DNA in heart, liver, and brain [120]. ROS produced during ethanol metabolism altered mitochondrial function [120,121]. Mitochondrially targeted vitamin E protected cerebellar granule cells from ethanol-cytotoxicity [122], suggesting that oxidative insults to mitochondria intimately influence cell fate. The integrity of mitochondria is intimately affected by membrane permeability that is regulated by a group of proteins called the mitochondrial membrane permeability transition pore (PTP). PTP regulates permeability to electrolytes, nucleotides and metabolic substrates, all of which are essential for ATP production. Although the composition of PTP remains uncertain, PTP is believed to be minimally composed of adenine nucleotide translocase, mitochondrial creatine kinase, voltage dependent anion channel, and cyclophilin D [123,124,125]. Oxidative or apoptotic stress renders excess opening of PTP, diffusing water and electrolytes across the mitochondrial membranes. Consequently, mitochondrial membrane potential (Δψm) collapses and oxidative phosphorylation of ATP fails [126]. Due to such a close relationship between PTP and Δψm, Δψm is often measured as a typical marker of PTP. 

**Figure 4:**
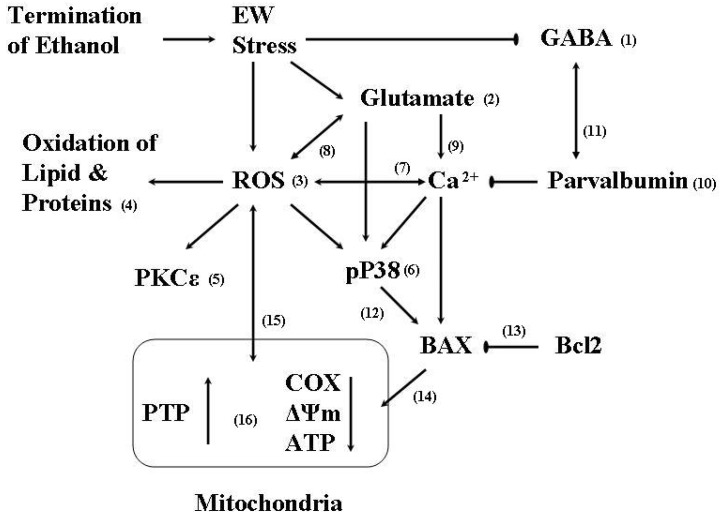
Oxidative signaling pathways of EW stress. The abrupt termination of excessive ethanol exposure downregulates inhibitory GABA (1) and upregulates excitatory glutamate (2) neurotransmittions. The excitatory stimulus of ethanol withdrawal (EW) provokes ROS generation (3), inducing the oxidation of lipid and proteins (4). ROS also trigger down-stream effectors such as the activation and translocation of PKCε (5), the phosphorylation of P38 (6), and an increase in the intracellular level of Ca^2+^ (7). A mutually enhancing effect (8) of ROS and glutamate may increase the activation of P38. The Ca^2+^ overload induced by glutamate (9) or by ROS is buffered by a Ca^2+^ buffering protein parvalbumin (10). Parvalbumin, located in GABAergic neurons, is downregulated in conjunction with EW-induced suppression of GABA neurotransmittions (11). The activation of P38 in turn activates BAX (12), which is inhibited by an antiapoptotic protein Bcl2 (13). Subsequently, BAX translocates to and inflicts mitochondria (14). Because mitochondria are the major source of ROS production, EW-induced excessive generation of ROS intimately impairs the integrity of mitochondrial membranes (15). Accordingly, the opening of PTP is increased, ΔΨm collapses, and the activity of cytochrome c oxidase (COX) is suppressed, all of which provoke ATP depletion (16). As net outcomes, neurons such as cerebellar Purkinje neurons are degenerated with behavioral impairments.

Studies suggest a deleterious effect of ethanol or EW on PTP. For instance, ethanol-induced cell death was prevented by cyclosporin A treatment (PTP inhibitor) [121,127]. Ethanol treatment resulted in excess PTP opening in mice lacking superoxide dismutase (endogenous antioxidant enzyme) [128], suggesting that a loss of endogenous antioxidant capacity may trigger PTP opening. We have demonstrated that EW provokes mitochondrial membrane swelling and Δψm collapse more severely than ethanol *per se* [44]. There are a few possible explanations on how EW targets mitochondrial membranes. First, EW provokes the excessive generation of free radicals which readily attack mitochondrial membrane components perhaps including PTP proteins. The oxidative modification of proteins alters the stability, conformation, activity, and function of the PTP proteins, resulting in PTP opening [129]. Second, excessive glutamate-induced neuronal excitation increases intracellular concentrations of Ca^2+^, which provokes PTP opening [37,124,130,131,132,133]. The interaction between Ca^2+^ and EW is shown such that a blockade of Ca^2+^ channels reduced EW signs [134] and EW-induced hyperanalgesia [135]. In addition, EW depleted the level of Ca^2+^ buffing protein (parvalbumin) in rats [12]. This effect of EW [12] is not surprising because parvalbumin is localized in GABAergic neurons [136] which are downregulated during EW. Third, protein kinases may mediate the mitopathic process such that oxidative stress induces translocation of PKCε to mitochondria, which in turn inhibits the electron transport chain and ATP production [137]. In our pilot study, a P38 inhibitor protected against EW-induced mitochondrial membrane swelling in HT22 cells. This protection may result from a counteracting effect of the P38 inhibitor on a proapoptotic protein bcl-2-associated x protein (BAX) [138]. P38 induces the translocation of BAX to mitochondria, thereby inflicting mitochondrial integrity [138]. This effect of BAX or P38 may be facilitated by EW-induced Ca^2+^. Two lines of studies support this notion. A synergistic interaction between intracellular Ca^2+^ and cytosolic BAX induces *in vitro* apoptosis [139] and Ca^2+^ mediates glutamate-induced P38 activation [140]. The multiple interactions between P38, BAX, oxidative stress, and mitochondria were well characterized in SH-SY5Y neuroblastoma cells: a pro-oxidant malonate activated P38 and a P38 inhibitor blocked malonate-induced BAX translocation to mitochondria [141]. Finally, EW may target key mitochondrial enzymes such as COX, thereby inflicting mitochondrial membrane integrity. COX catalyzes electron transfer at the terminal stage of the respiratory chain. The suppressed activity of COX has been found in a variety of neurodegenerative illnesses such as Alzheimer’s disease [142], suggesting a critical link between COX and CNS disorders. Moreover, this mitochondrial enzyme is also a target of oxidative stress such that nanomolar concentrations of nitric oxide rapidly inhibited the activity of COX in isolated mitochondria, brain nerve terminals, and cells [143]. Directly relevant to EW, repeated EW suppressed the activity of COX in the brain of young and aged rats [144]. Taken together, upon EW insults, multiple factors including ROS, Ca^2+^, and P38 simultaneously or sequentially interact with each other, inflicting the functional impairment of mitochondrial membranes (Figure 4). 

We have demonstrated that EW decreased the activities of total COX in rats [145] in a manner that was prevented by E2 treatment. Estrogens have substantial effects on mitochondrial function [146], particularly in the face of stress. Estrogen exerted protection against β-amyloid- or ROS-induced mitotoxicity [146]. In a rat model of oxidative stress, E2 prevented mitochondrial peroxidation induced by ovariectomy [147]. *In vitro* estrogen treatment to human neuroblastoma cells also protected against ATP depletion, ΔΨm collapse, and ROS generation induced by mitochondrial toxins [148] or H_2_O_2_ [116]. These mitoprotective effects of estrogen correlated with its antioxidant effects [119]. Furthermore, non-feminizing estrogens such as 17α-estradiol and ent-estradiol shared the ability of E2 to protect ATP production and ΔΨm maintenance [149]. In Wang's study [149], the ER antagonist ICI 182,780 failed to block the mitoprotection exerted by these estrogen analogues, suggesting that ER independent non-genomic mechanisms [150] mediate the protection. This non-genomic mechanism of E2 is supported by Alele and Devaud’s study [7] in which E2 and the neuroactive steroid pregnanolone rapidly mitigated EW-induced seizure activity. The non-genomic E2’s protection appears to occur at the mitochondrial level through modulating protein-protein interaction. For instance, E2 activates an antiapoptotic protein Bcl2 [151] which in turn inhibits the effect of a proapoptotic protein BAX on mitochondria. Given these findings, estrogen seems to play a role in alleviating oxidative or apoptotic burden in mitochondria, thereby protecting mitochondrial functions such as mitochondrial respiration [152]. 

As mentioned earlier, if pro-oxidants provoke PTP opening [129], antioxidants should inhibit the opening. Indeed, PTP opening was inhibited by vitamin E but was induced by H_2_O_2_ [153,154]. Based on these studies, we assessed antioxidant protection against PTP opening using three compounds which have ROS scavenging activities: E2, an antioxidant BHT, and an E2 analogue ZYC26. ZYC26 previously showed a 10-fold higher antioxidant potency than E2 in the HT22 cell model of EW [15]. ZYC26 contains an adamantyl group at the C2 position of A-ring and this structural configuration enhances the stability of the adjacent phenoxyl radical, which is an essential element of ROS scavenging activity [51,53,155]. We computed the magnitude by which these three compounds protect HT22 cells from EW-induced mitochondrial membrane swelling. Despite that ZYC26 more potently scavenged ROS than E2, all three compounds mitigated the mitochondrial membrane swelling with a similar degree of potency (data not shown). These observations indicate that other factors, in addition to the ROS scavenging activities of E2, also mediate protection against PTP opening. For instance, E2 may activate glutathione peroxidase which directly reduces membrane-bound lipid hydroperoxides [156,157]. It is also possible that E2 elevates the levels of endogenous antioxidants such as glutathione so that a favorable redox potential for an antioxidant environment is created [63]. Alternatively, because high levels of mitochondrial Ca^2+^ impede the regulatory function of the PTP proteins [124] and E2 induces mitochondrial tolerance to Ca^2+^ overload [158], E2 may increase a threshold of mitochondrial Ca^2+^ that is required to trigger PTP opening. At the very least, E2 appears to orchestrate multiple mechanisms in addition to an antioxidant action, effectively protecting mitochondria from EW. 

## 5. EW & Brain Aging

Alcohol is a commonly abused substance among elderly people [159,160,161]. Moreover, due to the long lasting nature of alcoholism, many alcohol dependent individuals experience aging in the course of alcoholism. Although many studies have investigated the interaction between age and ethanol [6,144], few studies have addressed brain aging associated with EW. An interaction between functional brain aging and EW was shown in a human study in which 70% of abstinent alcoholics suffered from aging-like neuropsychological impairments, including deficits in motor performance, short-term memory, and visuospacial performance [162]. Age-dependent effects of EW are also shown in animal studies in which EW provoked neuronal loss in the locus coeruleus in old male rats (29-30 months) but not in young rats (3-4 months) [163]. Thus, EW-induced neurobehavioral deficits may closely resemble psychomotor aging and these two variables (age and EW) may interact adversely with one another through common mechanisms. 

Both EW and aging are closely associated with oxidative stimulus. ROS are overproduced in aged subjects [164,165,166], and oxidative stress promotes age-related changes in cellular components [167,168,169,170]. The pro-oxidant nature of EW is particularly important in brain aging because it increases oxidative stress in the areas that are most vulnerable to aging, thereby facilitating age-associated disorders [171,172]. We have determined whether EW acts as a provoking stimulus for aging-like damage at the level of motor performance and cellular redox status. Ovariectomized female rats with or without E2 implantations, completed a diet program of 25-day ethanol exposure followed by 5-day EW/cycle, repeating three cycles. They were then tested for cerebellar-related motor performance and oxidative stress. Ethanol withdrawn rats displayed significant motor deficit and severe cellular oxidation at an earlier age than control-diet rats in a manner that was ameliorated by E2. These observations suggest that EW hastens brain aging and estrogen mitigates the problem. The age-associated motor deficit did not appear to be due to different amounts of ethanol intake or ethanol kinetics between ages. In general, old people consume a smaller amount of ethanol than young people even among alcoholic population [173]. Similarly for experimental animals, older rats consume less of an ethanol diet than young rats. In spite of the lesser ethanol intake, old rats had a higher blood ethanol concentration [174,175] and a slower ethanol elimination rate than young rats [176]. However, a higher blood ethanol concentration in old rats unlikely mediates their greater vulnerability to EW because the degree of EW-induced motor deficit did not correlate with blood ethanol concentrations. These findings suggest that the kinetics of ethanol unlikely contributes to the EW-induced motoric aging. 

P38 has been implicated in age-associated physiological or pathological conditions. While old men had a higher level of basal pP38 in skeletal muscles than young men, the opposite trend in age-associated pP38 levels occurred after strenuous exercise [177]. Mutant mice that formed the pathological aggregation of tau proteins had excess levels of pP38, which increased with age [178]. Pathological activation of P38 was shown in the brains of Alzheimer’s disease patients [179] and in the livers of aged rats after challenged with H_2_O_2_ [180]. Given this information, we tested the hypothesis that EW acts as an age-specific stressor to activate P38 in cerebellar neurons. Although the cerebellum contains multiple types of neurons, Purkinje neurons constitute the single output of all motor coordination in the cerebellar cortex [181]. Therefore, damage to these neurons inevitably provokes impairment of motor behavior and motor learning. For instance, cerebellar ataxic mice had a loss of Purkinje neurons but no change in the cerebellar granular neurons [182]. Purkinje neuron knock-out mice showed more severe motor deficit in a Rotarod test than granular neuron knock-out mice [183]. The ability of the connection (synaptic plasticity) between Purkinje neurons was lost by ethanol treatment [184]. In fact, a substantial loss of these neurons coincided with motor deficit in ethanol-withdrawn rats [9,57]. Using immunohistochemistry, we observed that pP38 positive Purkinje neurons were more populated in middle-age (15 months) EW rats than young (8 months) or older (19 months) EW rats. Similarly, EW-induced oxidation of mitochondrial proteins was more severe in middle-age rats (15 months) than other age groups (8 and 19 months). These findings suggest that the most vulnerable age to EW insults is not necessarily the old age and may vary depending upon target molecules of EW. As far as P38 is concerned, middle-age (12-15 months) was most vulnerable among the age categories tested (5-8, 12-15, and 16-19 months). In addition, the localization of pP38 in ethanol-withdrawn Purkinje neurons implies that P38 activation intimately interferes with the neuronal integrity. 

The susceptive neuronal response of middle-age rats to P38 activated by EW was coherent with behavioral manifestation in that EW-induced motor deficit was exacerbated by age, which began at 12-15 mo. A previous study also reported the vulnerability of middle-age groups to aging symptoms such that the onset of memory impairment occurred at the age of 12 months in female rats [185]. In a clinical study, brain changes resembling Alzheimer’s disease were most evident during the ages 50 to 59 years [186]. Multiple tasks involving cognitive and motor functions also declined during middle-age [187,188]. The susceptibility of middle-age rats may be the reflection of cellular and neuronal alterations associated with transition stress from young to old endogenous systems. The variety of alterations may collectively perturb the neuronal integrity, providing favorable conditions for P38 activation upon EW insults. In this scenario, animals a few months older than the middle age may develop adaptations and tolerances to the altered cellular and neuronal milieu and thus become more resistant to the EW stress. Alternatively, age-associated alteration may depend on specific organelles or molecules. For instance, when •O_2_^-^ and H_2_O_2_ were measured in mitochondrial fractions, the content peaked in middle-age rats [189]. In comparison, when total ROS content was measured in the whole-cerebellar lysates (not specific organelles), it peaked at an older age (19 months). The ROS results suggest that the vulnerable age at which maximum damage occurs may vary depending upon target molecules or organelles and the nature of stress.

Protective effects of E2 on age-associated motor deficit have been shown in studies where E2 administration improved the motor performance of 24-month-old female rats [190]. In contrast to these beneficial effects of estrogen, some studies reported adverse effects of estrogen on age-related phenomena such as decline in memory or cognition in humans [191] and in animals [192]. Among these reports, there seems to be a consistent agreement that estrogen treatment soon after ovariectomy produces beneficial effects on cognition, whereas a delayed treatment after ovariectomy has little or no beneficial effects. When treated with E2 at three months following ovariectomy, middle-aged rats learned a spatial memory task more quickly than vehicle-treated rats; however, when E2 treatment was delayed until 10 months after ovariectomy, no effects of E2 were observed [193]. Similar results were observed in studies of rhesus monkeys [191,194] and humans [195], suggesting that the neuroprotective effects of estrogen may require a certain treatment window. When implanted immediately after ovariectomy, we did observe that old, ethanol withdrawn rats treated with E2 behaved like young rats in motor performance, and showed similar cellular oxidation properties and hyperactivation of P38. 

## 6. Conclusions

Beyond classical mechanisms of the hyperactivation of excitatory and the suppression of inhibitory neurotransmissions, EW also induces the excessive generation of oxidative molecules which trigger the cascades of downstream signaling proteins such as protein kinases. The EW-induced ROS directly or indirectly attack mitochondria through the signaling protein kinases. Consequently, mitochondrial membranes swell, membrane potential collapses, and cytochrome c oxidases loses its activity, all of which contribute to mitochondrial pathology. When the brain is repeatedly exposed to such chaotic events, neurons are injured with behavioral consequences, the gravity of which is exacerbated at a vulnerable age. Estrogen may interfere with upstream oxidative pathways through its antioxidant activity, thereby protecting downstream effectors. Alternatively, E2 may simultaneously regulate oxidative pathways and signaling protein kinases, collectively executing protection. In addition to oxidative signaling mechanisms that were discussed in this review, numerous other pathways and complicated interactions between them are believed to mediate EW-induced brain injuries or E2’s protection. Unfortunately, it is difficult to differentiate each pathway to determine the degree to which EW or estrogen directly affects each step and how much is due to its effects on the others. It is our hope that this work contributes to a new insight into oxidative signaling pathways of EW and triggers further studies to identify molecular targets for better EW management. 
